# Treatment adherence amongst drug users attending public and private methadone maintenance clinics in a northern province of Vietnam

**DOI:** 10.1186/s13011-020-00271-9

**Published:** 2020-04-28

**Authors:** Tuan Anh Le, Giang Hai Ha, Mai Quynh Thi Le, Lien My Hoang Tran, Duyen Thanh Thi Pham, Ninh Hai Thi Tran, Giang Thu Vu, Long Hoang Nguyen, Hai Quang Pham, Cuong Tat Nguyen, Tung Hoang Tran, Kiet Tuan Huy Pham, Bach Xuan Tran, Carl A. Latkin, Cyrus S. H. Ho, Roger C. M. Ho

**Affiliations:** 1 National Institute of Hygiene and Epidemiology, Hanoi, 100000 Vietnam; 2 Institute for Global Health Innovations, Duy Tan University, Da Nang, 550000 Vietnam; 3 National Hospital for Tropical Diseases, Hanoi, 100000 Vietnam; 4 Center of Excellence in Evidence-based Medicine, Nguyen Tat Thanh University, Ho Chi Minh city, 700000 Vietnam; 5 Center of Excellence in Behavioral Medicine, Nguyen Tat Thanh University, Ho Chi Minh city, 700000 Vietnam; 6 Institute of Orthopaedic and Trauma Surgery, Vietnam – Germany Hospital, Hanoi, 100000 Vietnam; 7 Department of Lower Limb Surgery, Vietnam – Germany Hospital, Hanoi, 100000 Vietnam; 8 Institute for Preventive Medicine and Public Health, Hanoi Medical University, Hanoi, 100000 Vietnam; 9 Bloomberg School of Public Health, Johns Hopkins University, Baltimore, MD 21205 USA; 10 Department of Psychological Medicine, National University Hospital, Singapore, 119074 Singapore; 11 Department of Psychological Medicine, Yong Loo Lin School of Medicine, National University of Singapore, Singapore, 119228 Singapore; 12 Institute for Health Innovation and Technology (iHealthtech), National University of Singapore, Singapore, 119077 Singapore

**Keywords:** Adherence, Public methadone maintenance clinics, Private methadone maintenance clinics, Vietnam

## Abstract

**Background:**

Methadone maintenance treatment (MMT) has been proven to be effective in improving health status and the quality of life of illicit drug users. Due to the quick expand of methadone program, socialization through co-payment service is a critical to the success of it. In Nam Dinh, Vietnam, MMT has been used in public clinics and one private clinic. Such effectiveness of this treatment has been found to depend largely on adherence to treatment. This study aims to explore the compliance rate and its influencing factors among drug users between public and private clinics in Nam Dinh province, Vietnam.

**Methods:**

A cross-sectional study was conducted on 395 participants from January to September in 2018 in three MMT clinics in Nam Dinh, Vietnam. We applied the convenience sampling technique to recruit respondents. Data on socioeconomics characteristics, MMT adherence (measured by Visual Analogue Scale – VAS) and level of social/family support were collected.

**Results:**

43.3% of participants reported complete adherence to the MMT program during the time of research. Significant factors affect MMT adherence among illicit drug users including family income, history of drug rejections, concurrence in drug usage, far distance from MMT clinics, and having only peer. Patients in MMT private clinic had higher complete adherence than that of public MMT (OR = 1.82, 95% CI = 1.13; 2.94). Having contacts with peer drug users associated with a higher rate of incomplete adherence (OR = 2.83, 95% CI = 1.39; 5.73).

**Conclusions:**

The findings support the establishment of private MMT clinics alongside public ones, while further researches to determine the optimal dose and ways to reduce the impact of peer drug user’s influence are encouraged to be conducted.

## Background

Drug use disorder is considered to be one of the most serious public health problems globally [1]. The number of worldwide death cases caused by drug misuse has been increasing by more than 60% in the period 2000–2015 [2]. Drug misuse posed a threat to not only personal health but also socioeconomic problems, such as poverty and family breakdown [3]. Vietnam is not an exception, with the number of registered drug illicit users was 222,582 people in 2017 and 31,551 people were arrested involving in drug-related crimes in 2014 [4]. Moreover, injecting drug use caused more than 50% of all reported cases of Human Immunodeficiency Virus (HIV) [5]. To combat the drug use and reduce the HIV/AIDS among high-risk behavior community, methadone maintenance treatment (MMT) has been launched since 2008 [5, 6]. Several studies showed the benefits of MMT maintenance worldwide: reduced HIV transmission among drug injecting users and criminal activities caused by drug users [7]; helped reducing death rates, morbidity and social illness or being homeless [8–10].

In the Vietnamese context, MMT has been proved to be effective to reduce HIV transmission [11, 12]. The long-term effects of opioids (physical dependence and addiction) are reducing among patients having MMT treatment [13]. MMT increased the quality of life of both drug users and their families [14].

MMT is a long-term therapy and its effectiveness can be achieved with good adherence [15] because poor adherence reduces the positive effect of an intervention, which leads to health worsening and health care cost increasing [16]. Especially in MMT, failure to adherence is a risk factor for overdose causing by the changes of the central nervous system [17]. In China, the medication adherence rate has ranged from 11.8% at 8 years in Xi’an (14 clinics, 2006–2013) [18] to 26.57% at 5 years in Yunnan (1 clinic, 2007–2011). In Canada, a 15-year research found a very low rate of adherence to MMT among homeless adults with mental illness [10]. In Vietnam, the level of incomplete MMT adherence was various from the big city (from 8.3 to 17.7%) [11] to the mountainous setting (37.7%) [19].

Several scientific papers have identified the factors associated with MMT medication adherence. They were non-treatment factors such as sociodemographic characteristics (stable job, and monthly income), social support (family, children, and health staff support), and social stigma [11, 19, 20]; treatment-related factors such as e-health (mobile phone support) or methadone dose [21].

In Western countries, such as Canada, the United States or Europa, MMT is provided mostly by four main providers of MMT, including family physicians, multidisciplinary clinics, private clinics, and prisons [22, 23]. Meanwhile, in China or Vietnam, MMT is applied by public clinics [24, 25]. However, since 2015, to maintain and ensure the expand of the MMT program, Vietnam government is encouraged to establish private clinical model or socialization methadone treatment. Local provinces are responsible for finance the fees and some part of it is the co-payment from the patients (e.g., incidental supplies) [24].

Nam Dinh is one of the provinces that have the highest number of drug users in the North of Vietnam [26]. By the end of 2016, there were eight MMT clinics in the province, making it the 8th among 61 provinces of Vietnam with the most MMT clinics. Furthermore, Nam Dinh was the first province in Vietnam to establish a private MMT clinic in Vietnam. The Vietnamese government has started to involve the private health sector in providing MMT services as a preparation for the reduction and possible withdrawal of foreign funding that has long allowed the government to provide MMT to patients free-of-charge. There is no difference in the opening hours or payment between private and public MMT clinics and both establishments are under the same government control, including being granted, re-granted or withdraw the operation license for MMT treatment establishment, sending monthly reports to the local provincial agencies [27]. The establishment of private MMT clinics has sparked some debates on how those with drug use disorders, many from a financially disadvantaged background and without permanent jobs, would afford the cost of MMT and related services. Thus, understanding the level of adherence of MMT patients in two clinic models in Nam Dinh as well as the factors associated with adherence would bring valuable insights into the MMT situation in Vietnam and help identify possible areas for improvement. The aim of this study is to explore the medication adherence level among drug users enrolled in MMT clinics in Nam Dinh and determine the factors influencing adherence to treatment.

## Methods

### Study setting and subjects

A cross-sectional survey was used to collect data in Nam Dinh province from January to September 2018. The study was conducted at 3 outpatient clinics including Giao Thuy district health center (public model), Dai Dong private health facility (private model) and Giao Thuy Center for Social Evils Prevention (public model). The eligibility criteria for selecting MMT clinics were (1) following the guidelines of the Vietnamese Ministry of Health (MOH) in Methadone treatment [28]; (2) providing methadone treatment was at least 12 months.

The convenience sampling technique was used to recruit participants who fulfilled the following criteria: 1) at least 18 years old; 2) receiving Methadone treatment from above clinics; 3) giving consent to participate in the study; 4) being able to be communication with the data collection personnel. Participants, who were unable to answer the question during the recruitment process, were excluded from the research. In total, people agreed to participate in the study. According to the regulation of the Vietnam Government, the patients must go to the fixed clinics which they registered in advance. The proportion of patients in three health facilities were as follow: 49.4% (Dai Dong private health facility), 25.3% (Giao Thuy district health center) and 25.3% (Giao Thuy Center for Social Evils Prevention).

### Measure and instruments

Participants were invited to participate in 20-min face-to-face interviews. The data collectors were trained well to ensure data is collected timely and accurately. In order avoid social desirability bias, we did not invite local health staff to participate in data collection. We recruited MMT patients when they visited clinics for receiving treatment. The interview was processed in a private room with restricted access to ensure privacy and confidentiality. After introducing the purpose of the study, the advantages and disadvantages when participating in the study, participants were asked whether they want to join the research and the informed consent was given verbally. The participants did not receive any compensation when taking part in the study.

In the pilot survey, there were 20 people participating with various characteristics to examine the acceptability and reliability of the questionnaire. Only slight changes in wording were made to be suitable for a participant’s preferences and local culture. We developed a structured medication questionnaire with the following facts:

### Socioeconomic characteristics

The information including gender, age, marital status, education, occupation, monthly income was self-reported by participants.

### Adherence to methadone maintenance treatment services

We use the 100-point visual analog scale (VAS) to detect patients’ adherence. The scores of this scale range from 0 “incompletely adherence” to 100 “completely adherence”. The information on MMT duration and the number of times that participants missed doses in the last 30 days were also extracted from medical reports.

### Social/family support

To recognize the back from family and social, we inquired participants about whether they received the support during MMT duration, subjects that they need support the most, the most effective measure implemented to ensure dosage adherence and the kinds of support they feel necessary for complete adherence to MMT.

### Statistical analysis

We used STATA version 14 (Stata Corp. LP, College Station, United States of America) to analyze the data. A Chi-square test and a Mann Whitney test were used for analyzing demographic characteristics and social/family support of MMT clients. We proposed and explored a multivariate logistic regression model to identify factors associated with adherence to MMT service. We used a backward stepwise selection strategy that removed factors that had a p-value of log likelihood ratio test greater than 0.2. A value of *p* < 0.05 was perceived as statistical significance.

## Results

Table 1 shows the demographic and substance use characteristics of participants. Most of the participants (74.7%) fell in the age range 30–50 years old and had at least secondary school education (83.3%). In terms of marital status, the majority of participants were living with partners/spouses (77%). Risky behaviors of alcohol drinking and smoking were reported in the majority of respondents (53.4 and 81%, respectively). The average family income was 430.5 USD per month. There were significant differences in terms of age and employment status between completely and incompletely adherence groups.

**Table 1:** Demographic and substance use characteristics of participants

Characteristics	Completely adherence		Incompletely adherence		Total		***p***-value
	n	%	n	%	n	%	
**Age group**							
Under 30	10	5.9	38	17.0	48	12.2	0.01*
30–40	77	45.0	92	41.1	169	42.8	
41–50	61	35.7	65	29.0	126	31.9	
Above 50	23	13.5	29	13.0	52	13.2	
**Education**							
Less than secondary	30	17.5	36	16.1	66	16.7	0.38*
Secondary school	107	62.6	130	58.0	237	60.0	
More than secondary	34	19.9	58	25.9	92	23.3	
**Marital status**							
Single	20	11.7	47	21.0	67	17.0	0.05*
Living with partners/ spouses	141	82.5	163	72.8	304	77.0	
Divorced/widow	10	5.9	14	6.3	24	6.1	
**Employment**							
Unemployment	10	5.9	23	10.3	33	8.4	0.01*
Freelancer	49	28.7	90	40.2	139	35.2	
Blue collar worker/farmer	45	26.3	47	21.0	92	23.3	
Self-employed	11	6.4	17	7.6	28	7.1	
Others	56	32.8	47	21.0	103	26.1	
**Quintile monthly family income**							
First	35	20.5	45	20.1	80	20.3	0.65*
Second	37	21.6	45	20.1	82	20.8	
Third	29	17.0	52	23.2	81	20.5	
Fourth	38	22.2	44	19.6	82	20.8	
Fifth	32	18.7	38	17.0	70	17.7	
**Alcohol drinking**	88	51.5	123	54.9	211	53.4	0.50*
**Smoking**	139	81.3	181	80.8	320	81.0	0.90*
	**Mean**	**SD**	**Mean**	**SD**	**Mean**	**SD**	***p*-value**
Average family income (USD)	375.9	246.7	472.1	679.5	430.5	538.4	0.92^#^

*Chi square test, ^#^ Mann-Whiney rank sum test

The medication adherence among MMT patients is shown in Table 2. The complete adherence was reported in only 43.3% of respondents. Regarding missing dose, 90.9% of the patients reported not ever missed dose within the last 30 days. The median of the number of missed doses in the last 30 days was six (IQR = 2–15,5).

**Table 2:** Medication adherence among MMT patients

Characteristics	n	%
**MMT ever missed dose**		
Yes	36	9.1
No	359	90.9
**MMT adherence**		
Completely adherence	171	43.3
Incompletely adherence	224	56.7
**Reason for missing dose**		
Forgetting	8	12.3
Health reasons	7	10.8
Other	50	76.9
	**Median**	**IQR**
**MMT adherence VAS**	99	90–100
**Number of missed doses**	6	2–15.5

Table 3 presents the drug use status and MMT treatment duration of participants. Approximately two-thirds of the respondents had injected drugs (63.8%). The majority of patients reported no concurrent illicit drug use (94.2%). Most of the participants (95.4%) considered their quality of life to be ‘better’ since enrollment to MMT treatment. The average age of starting to use drug use was 26 years old (SD = 7.8). The mean MMT dose was 69. 2 mg (SD = 37.0) and the average MMT treatment duration was 3.3 years (SD = 2.2). There were statistically significant differences in terms of drug injection and concurrent illicit drug use between completely and incompletely adherence groups.

**Table 3:** Drug use status and MMT treatment duration of participants

Characteristics	Completely adherence		Incompletely adherence		Total		***p***-value
	n	%	n	%	n	%	
**Ever injected drugs**							
Yes	98	57.3	154	68.7	252	63.8	0.02*
No	73	42.7	70	31.3	143	36.2	
**Concurrent illicit drug use**							
Yes	1	0.6	22	9.8	23	5.8	< 0.01*
No	170	99.4	202	90.2	372	94.2	
**Quality of life since MMT onset**							
Better	164	95.9	213	95.1	377	95.4	0.70*
Unchanged	7	4.1	11	4.9	18	4.6	
	**Mean**	**SD**	**Mean**	**SD**	**Mean**	**SD**	***p*-value**
**Age of onset of drug use**	26.5	8.1	25.5	7.6	25.9	7.8	0.25^#^
**MMT duration (year)**	3.2	2.2	3.3	2.2	3.3	2.2	0.38^#^
**MMT dose (mg)**	72.9	43.7	66.4	30.7	69.2	37.0	0.29^#^

*Chi square test, ^#^ Mann-Whiney rank sum test

Table 4 reveals the characteristics of social support in MMT treatment. A large portion of participants had received support in MMT treatment from their family (77.7%) and health workers at MMT facility (41.3%). These two support providers were also perceived as the most needed sources of support by a high percentage of participants (77.7 and 36.5%, respectively). The majority of patients reportedly considered remembering the treatment schedule by themselves to be the most effective way for MMT adherence (73.2%) and no additional type of support needed to improve adherence (75.2%).

**Table 4:** Social support in MMT treatment

Characteristics	Completely adherence		Incompletely adherence		Total		***p***-value
	n	%	n	%	n	%	
**Current support provider**							
None	41	24.0	47	21.0	88	22.3	0.48*
Health workers at MMT facility	85	49.7	78	34.8	163	41.3	< 0.01*
Other health workers	4	2.3	4	1.8	8	2.0	0.73^¢^
Relatives in family	130	76.0	177	79.0	307	77.7	0.48*
Peer in MMT	22	12.9	45	20.1	67	17.0	0.06*
Neighbors/other acquaintances	9	5.3	7	3.1	16	4.1	0.29*
**Perceived needed support provider**							
None	35	20.5	45	20.1	80	20.3	0.93*
Health workers at MMT facility	77	45.0	67	29.9	144	36.5	< 0.01*
Other health workers	9	5.3	7	3.1	16	4.1	0.27*
Relatives in family	131	76.6	176	78.6	307	77.7	0.64*
Peer in MMT	26	15.2	27	12.1	53	13.4	0.36*
Neighbors/other acquaintances	10	5.9	7	3.1	17	4.3	0.19*
**Perceived most needed support provider**							
None	46	26.9	53	23.7	99	25.1	0.46*
Health workers	125	73.1	171	76.3	296	74.9	
**Perceived most effective type of support**							
Self-timer (clock, phone, etc.)	19	11.1	39	17.4	58	14.7	< 0.01*
Relatives support	2	1.2	20	8.9	22	5.6	
Remember by yourself	144	84.2	145	64.7	289	73.2	
Other	6	3.5	20	8.9	26	6.6	
**Perceived additional type of support to improve MMT adherence**							
None	140	81.9	157	70.1	297	75.2	0.01*
Methods to improve treatment	3	1.8	14	6.25	17	4.3	0.03*
Job search guidelines	10	5.9	18	8.0	28	7.1	0.40*
Financial support	26	15.2	50	22.3	76	19.2	0.08*

*Chi square test, ¢Fisher exact test

Table 5 demonstrates the health service utilization characteristics of the respondents. Half of the respondents used the public facility (50.6%). A large portion of respondents considered their distance to MMT facility to be near (46.1%) or of reasonable distance. The estimated average distance was 4 km (SD = 2.8 km). There were significant differences in terms of MMT model and estimated distance to MMT facility between completely adherence and incompletely adherence group. Regarding satisfaction with daily MMT service, the majority of participants reported being satisfied (78.7%).

**Table 5:** Health service characteristics

Characteristics	Completely adherence		Incompletely adherence		Total		***p***-value
	n	%	n	%	n	%	
**MMT model**							
Private facility	96	49.2	99	50.8	195	100.0	0.02*
Public facility	75	37.5	125	62.5	200	100.0	
**Estimated distance to MMT facility**							
Near	89	48.9	93	51.1	182	100.0	0.01*
Reasonable	77	41.9	107	58.9	184	100.0	
Far	5	2.9	24	10.7	29	100.0	
**Satisfaction with daily MMT service**							
Very satisfaction	8	40.0	12	60.0	20	100.0	0.47^¢^
Satisfaction	139	44.7	172	55.3	311	100.0	
Medium	24	39.3	37	60.7	61	100.0	
Dissatisfaction	0	0.0	3	100.0	3	100.0	
	**Mean**	**SD**	**Mean**	**SD**	**Mean**	**SD**	***p*-value**
**Distance to MMT facility (km)**	3.8	2.6	4.1	2.9	4.0	2.8	0.18^#^
**MMT service satisfaction**	8.6	1.0	8.6	0.9	8.6	1.0	0.75^#^

*Chi square test, ^¢^Fisher exact test, ^#^ Mann-Whiney rank sum test

Table 6 describes factors associated with MMT adherence. Participants with monthly family income in the third quintile (OR = 2.02, 95% CI = 1.13; 3.62), had history of drug rejections (OR = 1.75, 95% CI = 1.06; 2.88), engaging in concurrent drug use (Coef = − 27.66, 95%CI = 3.40; 224.78), receiving only peer support in MMT treatment (OR = 2.97, 95% CI = 1.46; 6.06) and living further away from MMT facility (OR = 8.82, 95%CI = 2.09; 37.19) were significantly more likely to incompletely adhere to MMT. Being employed and in the fifth (lowest) quintile of average monthly income were found to be positively associated with perceived adherence on the VAS scale. In contrast, participants, who engaged in concurrent illicit drug use, received only peer support in MMT, lived further away from MMT facility, had significantly lower VAS adherence scores. Patients of the public MMT model were found to have a higher chance of missing dose, while respondents living at a reasonable distance from the MMT facility were less likely to miss a dose.

**Table 6:** Associated factors with MMT adherence

	Incomplete adherence		Adherence VAS		Ever missed dose	
	OR	95% CI	Coef.	95% CI	OR	95% CI
**Marital status** (Living with partners/spouse vs single)	0.58*	0.33; 1.01	3.64**	0.38; 6.90	2.10	0.77; 5.74
**Occupation** (vs Unemployment)						
Self-employed			3.64*	−0.51; 7.80		
Blue collar worker/farmer			6.41***	1.91; 10.91		
Others			6.33***	1.85; 10.81		
**Quintile monthly family income** (vs First)						
Third	2.02**	1.13; 3.62				
Fifth			2.63	−1.16; 6.43		
**Smoke** (Yes vs no)					4.31*	0.99; 18.74
**History of drug injections** (Yes vs no)	1.75**	1.06; 2.88	−2.28	−5.34; 0.78		
**Concurrent illicit drug use** (Yes vs no)	27.66***	3.40; 224.78	−14.56***	−20.15; −8.98		
**MMT dose** (mg)	0.99**	0.98; 1.00	0.04*	−0.01; 0.08		
**Receiving support in MMT service** (Yes vs no)						
None	0.42***	0.22; 0.78	3.48*	−0.26; 7.21		
Health workers at MMT facility	0.28***	0.16; 0.49	5.28***	1.96; 8.60		
Commune health workers	0.24	0.03; 1.73	11.08*	−0.56; 22.72		
Peer	2.97***	1.46; 6.06	−4.00*	−8.02; 0.02		
**Most effective manner to not miss dose** (Remember by yourself vs Self-timer)	0.39***	0.22; 0.69			0.57	0.26; 1.23
**MMT model** (Public vs Private)	1.70**	1.06; 2.73	−3.55**	−6.45; −0.64	2.54**	1.21; 5.34
**Distance to MMT facility** (vs Near)						
Reasonable	1.98**	1.12; 3.50	−3.31**	−6.25; −0.37	0.52*	0.24; 1.12
Far	8.82***	2.09; 37.19	−7.74***	−13.10; −2.39		
**Distance to MMT facility (km)**	0.90*	0.80; 1.01				

*** *p* < 0.01, ** *p* < 0.05, * *p* < 0.1

## Discussion

Although MMT treatment among drug users in Nam Dinh is an important effects to reduce opioid use, the rate of MMT non-adherence in this study was 43.3%. Our research also highlights the positive effects of private clinics compared with public ones and the negative effect of peer-support to MMT treatment. The results of this study could be used to suggest possible adherence policies for MMT patients in Nam Dinh province.

In this study, we found that the level of MMT adherence in Nam Dinh province was 43.3% over 9 months. This finding was similar to previous research in Ho Chi Minh anh Hai Phong city, which are two big cities in Vietnam (42.1%) [11] and lower than that of Tuyen Quang, a mountainous province with the geographical barrier (34.4%) [21]. Compared with other research, the situation was the same with that in London (42%) and lower than that in some provinces in China with the adherence rate from 11.8 to 25.8% [18, 29]. The rate of MMT adherence is different across countries and regions, thus, there is a need to support drug users not only in pharmaceutical treatment but also in mental health treatment to increase the adherence and ensure MMT retention [11, 30].

Multilevel significant predictors of MMT adherence including socio-demographic (age, employment, and income), health care (MMT clinics model, receiving support in MMT service), self-help and other substance use disorder (self-timer, history of drug injection, concurrent illicit drug use), and medication use (MMT dose). Older age was a positive factor to completely adherence, and higher adherence VAS scale. Studies from Western and Eastern countries have also confirmed that older patients had better outcomes and retention in MMT programs meanwhile younger ones are the most likely to drop out substance use disorder treatment [31–34]. This can be explained by the increase in health concern and dissatisfaction with the unhealthy lifestyle among older people [33–35].

Moreover, our finding that distance to MMT clinics was a contributed factor to incomplete adherence. According to MoH requirement, they have to present at the clinics daily and take it with the presence of health staff, thus, when the MMT facilities are too far from their house, they may have to spend half-day for traveling to the MMT clinics and have no time to work [36]. The burden of traveling can be a barrier that affects the outcome of the MMT program, especially, in the mountainous and low-income areas of Vietnam where public transport is not covered.

In addition, people, who had a job (worker, or farmer) and higher monthly family income, had better adherence to MMT compared with those who were unemployed and had low income. This can be explained by the co-payment for methadone in Vietnam. At public MMT clinics, the current fee that is applied about 0.5 USD/patient/day that about 50% of the unit cost [37, 38]. In a recent study, a high level of current patients was willing to pay 32 USD/month. However, this was supported by some demographic factors such as income or education level [12]. Thus, socio-demographic factors should be carefully taken into consideration to maintain MMT after the funding donors are withdrawing from the program, which can ensure the equalities among patients who treated with MMT.

Regarding health care, a previous study in MMT service found that patients without support had lower adherence rate compared with those with support. Our result was in line with other studies, which showed that positive support from MMT providers could increase adherence [39, 40].

In Vietnam, there have been three MMT models: drug rehab center, public clinic, and private clinic. Notably, our study shows that the patients in MMT private clinics had higher complete adherence than the MMT public ones. Both kinds of clinics are under the control of the Vietnam Government and have the same opening hours (during office hours and 7 days a week). For patients in the rehabilitation center, all the fees are covered by the government. With public and private clinics, the government covers methadone fee and patients are required to copay for other services such as consulting or medical examination [41]. Normally, in Nam Dinh, drug users co-pay under the half US dollar a day for MMT treatment in both private and public MMT clinics [42, 43]. Thus, the higher rate of adherence among patients in MMT private clinic compared with that of public establishments could be explained that the private clinic may be less crowded, so it can offer higher quality service with the shorter waiting time. Consequently, the experience of patients may enhance and encourage them to follow the treatment.

With the withdrawal of international funding donors and restriction of budget, the Vietnam government will face difficulties in covering the full cost of all MMT clinics as well as in providing human resources for treatment. Therefore, the involvement of the private health sector would shoulder the financial and resource burdens, ensuring the availability of MMT services for those in need. On the other hand, subsidization for those of disadvantaged financial background would be necessary. As MMT was a long-lasting treatment, the cost of treatment can be a burden to the patients’ families [44, 45]. In essence, it is suggested that the most appropriate model for Vietnam would be the public-private model [46].

In this study, we also found that the adherence among MMT clients was lower in the group having a history of drug injection or still used a drug when being treated with methadone. A higher level of psychological distress [47] and/or the contact with peer drug users, [18], which are considered an unproductive factor. The similarity between peers regarding drug use has been found in several studies [48, 49]. Peers have influence on one’s individual behavior and that is the outcome of socialization [49]. This result shows the necessity for mental health consultant or treatment for MMT. Moreover, to reduce the negative impact of peers, eHealth intervention should be used to reduce opioid dependence [50].

Furthermore, the proportion of MMT smokers and MMT alcohol dependences, who were MMT non-adherence, was 80.8 and 54.9%, respectively. MMT patients could suffer smoking-related and alcohol-related diseases [51]. This phenomenon could be explained by the stigmatization when Vietnamese consider opiate absence as a priority [52]. This result suggests that tailored mental counselling for smoking cessation and quit drinking alcohol should be applied in MMT clinics [52].

Our result was along the same lines with some systematic review and randomized control trials research, that found out MMT dose was the main factor in MMT adherence [53–55]. Thus, there is a need to identify a suitable dose, maybe for each patient, to ensure the outcome of MMT [55].

The study is subjected to certain limitations. First, because of the cross-sectional nature that recorded data at one point in time, it would be difficult to reflect the casual association between risk factors and outcomes [56]. Second, the recall bias and social desirability response bias can be caused due to the self-reported data was. Third, the respondents were conveniently recruited may limit generalization to all populations. However, the results of this study still can be used as evidence for identifying risk factors in MMT adherence. In addition, due to the difference in the background of patients attending public and private clinics, there could be bias when comparing the MMT adherence of two clinic models. In addition, in this study, we employed stepwise selection strategies to produce the reduced model, which might result in bias in parameter estimation and the increased chance of a Type 1 error [57]. Finally, some associated factors such as the support of digital medicine or other psychiatric comorbidity or some services-related factors were not considered in this study. Therefore, more studies are required to meet the knowledge gap for improving the reliability and efficacy of the MMT program in Vietnam.

## Conclusions

In conclusion, the proportion of MMT adherence among drug users in Nam Dinh was similar to that of some countries worldwide. Beside some influencing factors such as socio-demographics, concurrent illicit drug use, and medication use, two factors should be received careful attention were MMT clinic model and the effect of peer drug users to MMT adherence. Further studies should be taken in Vietnam to evaluate the effectiveness of different MMT models to identify the most suitable one as well as to explore in more detail the factors supporting MMT adherence.

## Data Availability

The datasets used and/or analysed during the current study are available from the corresponding author on reasonable request.

## Abbreviations

AIDS: Acquired Immune Deficiency Syndrome
HIV: Human Immunodeficiency Virus
MMT: Methadone maintenance treatment
VAS: Visual analog scale
